# Role of Spirituality in Mental Health in Nepal

**DOI:** 10.31729/jnma.5110

**Published:** 2020-06

**Authors:** Anita Poudel

**Affiliations:** 1 Nepalese Army Institute of Health Sciences, Sanobharyang, Kathmandu, Nepal

The World Health Organization (WHO) defines health as ‘a state of complete physical, mental, and social well-being and not merely the absence of disease or infirmity’.^1^ WHO defines mental health as ‘a state of well-being in which the individual realizes his or her own abilities, can cope with the normal stresses of life, can work productively and fruitfully, and is able to make a contribution to his or her community’.^1^ From the time being, spirituality has played an important role in mental wellbeing.Globally acknowledged concept of spirituality involves belief and obedience related to God.Spirituality involves abstract ways such as searching for the meaning of life and a sense of connectedness to the universe.^2^ It is associated with markedly reduced mental illness and, is linked with higher mental health status.^3^

In the world where the majority of the population (90%) is involved in some form of religious and spiritual practice,^4^ around 450 million people currently suffer from various mental illnesses making it leading causes of ill-health and disability worldwide. One in four people in the world is affected by mental disorders at some points in their lives.^5^ In a country like Nepal where the day starts with prayers of God and every new work commences in the name of God, mental illnesses such as depression, bipolar disorders, anxiety, and various other disorders affect 13.2% of adults and 11.2% of children. Around 11% of adults and 9% of children commit suicide every year.^6^

The importance of spirituality in mental health is now widely accepted. Mental health has two dimensions- the absence of mental illness and the presence of a well-adjusted personality that contributes effectively to the life of the community. Essential features of mental health include being responsible, flexible, socially active, being wise, and able to appreciate harmonious relationships to self and others including nature and God. As a result, lack of spirituality disturbs interpersonal relationships leading to the genesis of psychiatric disturbances. For instance, depression commonly involves a lack of interest in religion whereas schizophrenia involves distorted religious practices. Moreover, the spiritual background of the patient plays a key role in the diagnosis and management of psychiatric disturbance.^2^ Religious practices are linked with increased hope and self-esteem along with a greater sense of meaning in life.^7^ Many psychiatrists, therefore, now believe that religion and spirituality are important in the life of their patients.^2^

The innovation which involves the change in regional cerebral blood flow and other metabolic parameters has opened the door for a more objective study of spiritualism in all its dimension. Through this technique, it is now possible to map brain activity associated with emotion and imaging the mind.^8^ Accordingly, the World Psychiatry Association, American Psychological Association, American Psychiatry Association, and Royal College of Psychiatrists all have included sections dedicated to religion and spirituality.^9^ Kristeller studied for the first time to investigate the feasibility and impact of taking a spiritual history. The study included 118 consecutive out-patients of 4 oncologists and they were alternately assigned to either usual care or usual care plus a spiritual history. The study, after three weeks, showed less depressive symptoms, a better quality of life, and a greater sense of interpersonal caring in patients from whom the spiritual history had been obtained as compared to the control group. Another study showed that it was extremely or somewhat important to discuss spiritual issues when counseling for mental disorders.^10^ Therefore, The World Psychiatric Association on the proposal of Lukoff et al. established a section on psychiatry and religion on DSM4, V 62.89 (Diagnostic and Statistical Manual of Mental Disorders, fourth edition) that includes three categories- normal religious and spiritual experiences; religious and spiritual problems leading to mental disturbances; and mental disturbances with a religious and spiritual context.^2^

People following spiritualism have reported tremendous positive feelings and a better capacity to deal with negative life events and stressors. Individuals with imperious religious faith report higher levels of satisfaction, greater personal happiness, and fewer negative consequences of traumatic life events.^11^ A couple of case histories reported by Sims better highlights the importance of spiritualism. In the first case, Jim who was suffering from Korsakov's psychosis was so deteriorated that he mistook his wife for a hat. In the hospital, he was considered as a desolate individual; however, he was normal in the chapel. He would partake Holy Communion in absolute concentration and attention. Also, he did not show any signs of illness during that period. The second case was of a chronic schizophrenic patient who used to hear his voice commanding him to jump out of the window. His simple devout mother had taught him to resist the voice by praying to God. Unfortunately, on one occasion he lost his life by being too late to pray.^12^

A scale that determines the importance of religion to people was developed by Ashby Wills who was a professor at Albert Einstein College of Medicine.^13^ The scale which was administered to 1182 children in New York found that religiosity kept children from smoking, drinking, and drug abuse. In the 10-year follow-up study done by Miller on depressed mother and their offspring,it was reported that maternal religiosity was protective against mental illness especially depression in offspring. It was also associated with low substance abuse in offspring.^14^ At the University of New Mexico, Scott Tonigan followed-up 226 patients of alcohol dependence and found that spirituality determines behaviors such as honesty and responsibility which in turn promoted alcohol abstinence.^15^ In an epidemiological study done in Britain, researcher Brown and Prudo found that going church protected from depression.^16^ Kerkoff studied in detail on suicide in the Netherland where he found that a religious revival declined the rate of suicide.^2^ A multicentric study conducted in the Department of Psychiatry, Christian Medical College showed that those patients suffering from schizophrenia who spent more time on religious activities tended to have a better prognosis on two years and five years follow-up. The above reports strongly suggest that religious beliefs and practices of psychiatric patients should be given importance.^17^

Nepal, being a predominantly Hindu country comprises a majority of people following spiritualism. As a result, many behaviors related to spiritualism are in practice. Behaviors such as early morning rise, praying, going on pilgrimage, intermittent fasting, listening to music and bhajan, and meditation can promote solace and mental health through peace and developing hope and positive thinking.^18^ Morning light is known to be a powerful cue for shortening the circadian cycle of the biological clock, which is naturally longer than 24 h in most humans. Also, light exposure in the morning has been reported to be effective for patients with depression and seasonal affective disorder.^19^ Waking up early morning increases the academic performance of the students.^20^ Many studies explain the positive effects of fasting on promoting mental health.It is reported that fasting improves self-esteem to a greater extent, which bestowsin maintaining a positive mental status. Fasting also has shown to reduce symptoms of anxiety and depression and improve social functioning.^21^ Listening to bhajan and yoga music greatly improves attention and concentration as a part of mental well-being.^22^ Meditation leads to metamorphosis of brain structure to emit positive emotions. The EEG records indicate that meditation can even tame the amygdala enabling the individual to be less shocked, flustered, or angry. Transcendental meditation promotes increasing degrees of orderliness, integration, and coherence in the brain leading to a unique style of brain functioning. The enlightened people maintain a low level of excitation, which has a calming effect on the mind.^10^ Among adults with a sustained personal spirituality over 5 years, MRI findings show cortical thickness in those regions of the brain (parietal, occipital, precuneus) to show cortical thinness in people with recurrent depression. In a related study involving EEG, posterior high amplitude alpha was identified in people with a strong personal spirituality who have recovered from depression. People tendencies such as a feeling of happiness, joyousness, and hope for the future are related to spirituality. Religious beliefs increase resistance against calamities, and thus help preserve physical and mental health, prevent the infliction of diseases, and finally promote hopefulness.^23^

**Way Forwar**d

The interrelationship between spirituality and mentalhealth have been known for decades. Holy books such as Bhagwat Gita, Tripitaka, Bible, and Quran can be profitably used to help the patient to cope with a life situation. Behavioral changes such as early morning rise, praying, going on pilgrimage, intermittent fasting in name of their preferred God, listening to peaceful music and bhajan, meditation, and many other activities related to spirituality might help in improving mental health. When positive thoughts and spirituality have a lot of positive benefits why not think about spirituality in mental health.

## Conflict of Interest:

**None.**
